# EDITOR’S MESSAGE / MESSAGE DE LA RÉDACTION

**DOI:** 10.29173/jchla29776

**Published:** 2024-04-01

**Authors:** Megan Kennedy

Welcome to the JCHLA/JABSC Spring Issue! In this issue, we have several research articles on topics related to the inclusion of librarians in knowledge synthesis grant funding and the scale of involvement of librarians in systematic review publications. There is also a scoping review discussing health sciences librarians' instructional engagement in continuing education. We have two column pieces that provide descriptions of recent CHLA/ABSC initiatives: the first column describes results from the EDI survey conducted by the Equity, Diversity, and Inclusion (EDI) Task Force; the second column gives an overview of the CHLA/ABSC Leadership Institute. Finally, we have two book reviews related to data-driven decision making and consumer health informatics and a product review on the Systematic Review Accelerator Deduplicator tool.

In addition to some wonderful content, we have an exciting announcement for our readers. Registration for the CHLA/ABSC 2024 Conference in Winnipeg is now open! If you are submitting your work to the CHLA/ABSC Conference, we also invite you to consider submitting your work to JCHLA/JABSC. Writing up your research article or program description into a full-scale article provides important detail and context for those interested in your work. You can read more about requirements for any potential submissions in our Author Guidelines.

**Megan Kennedy**

JCHLA/JABSC, Editor

Email: editor@chla-absc.ca

Bienvenue au numéro du printemps du JABSC/JCHLA! Dans ce numéro, nous présentons plusieurs articles de recherche sur des sujets liés à l’implication des bibliothécaires dans le financement de synthèse des connaissances ainsi que dans la publication de revues systématiques. On retrouve également un examen de la portée sur l'engagement pédagogique des bibliothécaires en sciences de la santé dans la formation continue. À cela s'ajoute deux rubriques décrivant les initiatives récentes de l'ABSC / CHLA : la première présente les résultats de l'enquête EDI menée par le groupe de travail sur l'équité, la diversité et l'inclusion (EDI) ; la deuxième donne un aperçu de l'Institut de leadership de l'ABSC / CHLA. Enfin, nous terminons avec deux revues de livres, l’une sur la prise de décision fondée sur les données et l’autre sur l'informatique de santé grand public, ainsi qu'une revue de produits sur l'outil Systematic Review Accelerator Deduplicator.

En plus de ce contenu exceptionnel, nous avons une annonce passionnante pour notre lectorat. La période d'inscription à la conférence CHLA/ABSC 2024 à Winnipeg est maintenant commencée! Si vous soumettez votre travail au congrès de l'ABSC / CHLA, nous vous invitons également à envisager de soumettre un article au JASBC/ JCHLA. La rédaction d'un article de recherche ou d'une description de programme sous la forme d'un article complet, fournit des détails et un contexte important pour ceux qui s'intéressent à votre travail. Pour en savoir plus sur la politique éditoriale en vue d'un éventuel article, consultez la page web sur les directives aux auteur-es.

**Megan Kennedy**

JABSC/JCHLA, Éditrice

Courriel: editor@chla-absc.ca

